# Nano fertilizer synergist effects on nitrogen utilization and related gene expression in wheat

**DOI:** 10.1186/s12870-023-04046-9

**Published:** 2023-01-12

**Authors:** Min Yang, Chengwu Dong, Yan Shi

**Affiliations:** Dryland-Technology Key Laboratory of Shandong Province, College of Agronomy, Qingdao Agricultural University, Qingdao, 266109 China

**Keywords:** *Triticum aestivum L.*, Nano materials, Nitrogen accumulation, Nitrate transporter, Glutamine synthase

## Abstract

The application of nano materials is one of the current hot spots in agricultural production. The aim of this work was to evaluate the effects of different nano fertilizer synergists on nitrogen (N) utilization and related gene expression in wheat. The experiments were carried out in pot and field conditions at the West-Coast Economic New Area experimental base and Greenhouse of Qingdao Agricultural University. Seven treatments were set up: CK (compound fertilizer), T1 (compound fertilizer + 0.3% nano carbon synergist), T2 (compound fertilizer + 0.3% nano calcium carbonate synergist), T3 (compound fertilizer + 0.3% composite nano synergist), T4 (70% compound fertilizer + 0.3% nano carbon synergist), T5 (70% compound fertilizer + 0.3% nano calcium carbonate synergist), T6 (70% compound fertilizer + 0.3% composite nano synergist). The results showed that compared with CK, the N accumulation of T1, T2, T3, T4, T5 and T6 increased by 40–50%, 30–40%, 55–65%, 20–30%, 15–20% and 30–40%, respectively; and the N use efficiency increased by 12–19%, 9–18%, 16–22%, 5–17%, 4–16% and 10–20% respectively. And the gene expression levels of *TaNRT2.2*, *TaNRT2.3*, *TaGS1* and *TaGS2* in the treatments with synergistic phosphate fertilizer were significantly higher than those in the CK. The application of nano fertilizer synergist can significantly improve N accumulation, N use efficiency, and promote the expression of genes related to N transport and metabolism.

## Introduction

Nitrogen (N) is one of the essential elements for crop growth, which can regulate many processes of wheat (*Triticum aestivum L.*) growth and development. The application of chemical fertilizers plays an important role in optimizing yield and quality of crops. According to statistics, the domestic application of chemical fertilizers accounted for nearly 40% of the World's application in 2011, and has been growing [1]. From January to May 2022, the output of agricultural N, phosphorus (P) and potassium (K) fertilizers in China has reached 23.2 million tons. If the application of chemical fertilizers is seized, the global crop production output could be reduced by about 50% [2]. However, since the 1990’s, the impact of increasing the amounts of fertilizers on improving crop yield has been no longer significant, and the fertilizer utilization efficiency is relatively low. The average N utilization rates of wheat in China in 1990–1999, 2000–2005 and 2006–2010 were 35, 28.2 and 34%, respectively [3, 4]. The problem of high amounts of fertilizer and low utilization efficiency in modern crop production is prominent [5]. Overapplication of chemical fertilizers is also associated with multiple issues such as decline of economic returns and environmental pollution. Therefore, improving chemical fertilizer efficiency and sustaining high yield and quality of crops is one of the main challenges in agricultural development.

Wheat is one of the important food crops. About 35% ~ 40% of the World's population utilizes wheat as the key food source [6]. China is a major wheat producing country, and its wheat output accounts for about 17% of the total global annual wheat production. Therefore, understanding how to improve the yield and quality of wheat can better meet the growing food demand of humanity and promote social and economic development. Nitrogen is present in the soil in the form of ammonium (NH_4_^+^), nitrate (NO_3_^−^) and organic compounds. About 92–98% of the total N in the soil is in organic form as soil organic matter. Only 2–8% of the total N in the soil is in mineral form (i.e. NH_4_^+^ and NO_3_^−^). However, only the mineral forms of N are readily available for plant uptake, while the organic N has to be mineralized into ammonium and nitrate for root uptake [7–9]. Nitrate is the main N source of higher plants. The absorption and transport of NO_3_^−^ in plants are achieved through NO_3_^−^ transporters (NRT) [10]. Nitrogen assimilation is accomplished by glutamine synthase (GS), which is a key catalytic enzyme to convert inorganic N into organic N and is crucial in N utilization. The GS gene overexpression can promote the expression of NRT family genes to enhance the absorption, assimilation and accumulation of N in crops [11]. Some studies have shown that the application of nano fertilizers to the soil can reduce the loss of NO_3_^−^ via leaching and of NH_4_^+^ via volatilization [12]. Nano fertilizers have shown potential to reduce the environmental pollution [13] and also improve the efficiency of N utilization for enhancing wheat yield and quality.

Nano materials are a kind of ultra-fine materials with special properties such as surface effect and small-size effect [14]. With the continuous development of nano science and nano technology, nano carbon has attracted great attention in agriculture. In recent years, nano materials have been gradually applied to agriculture and become the current research hotspot. In some studies, nano carbon was applied to crops as fertilizer synergist. Nano carbon has advantages in gene regulation, cell infiltration and fertilizer slow release [15]. The fertilizer added with nano carbon can improve the solubility and dispersion of nutrients in soil [16], increase the nutrient uptake by crops from fertilizer, reduce the loss of fertilizer in soil and improve the utilization rate of fertilizer; thus promoting the growth, development and yield of the crops, increasing the yield, and also reducing the environmental pollution, which is beneficial to the sustainable development of agriculture [17]. Studies have shown that the application of nano carbon can significantly improve utilization efficiency of N fertilizer and P fertilizer of tuber mustard (*Brassica juncea var. tumida*) [18]; and the combined application of nano calcium carbonate, humic acid and organic fertilizer can promote the absorption of N, P and K by peanut (*Arachis hypogaea Linn.*) plants, meet the nutrient requirement for the growth and development of peanut during the flowering needle stage, and help improve the fruit setting rate and yield of peanut [19]. At present, there are few studies on composite nano synergist in agriculture, and the mechanism of nano carbon synergist, nano calcium carbonate synergist and composite nano synergist is not completely clear, which needs further study [20].

The advantages of nano materials in agricultural production and fertilizer slow release have brought new opportunities for agricultural development. The application of nano fertilizers in agriculture can significantly improve the efficiency of agricultural investment, and also provide an important way to maintain the sustainable development of agricultural ecosystem [21]. Therefore, this experiment—by adding nano carbon synergist, nano calcium carbonate synergist and composite nano synergist—aims to explore whether the addition of different nano fertilizer synergists can promote wheat N utilization and related gene expression.

## Materials and design

### Experimental materials

The wheat variety Yannong 999 was used in the experiments, which was approved by the Yantai Academy of Agricultural Sciences (approval No. 2016012). It is a semi-winter wheat and has good cold resistance in winter, strong tillering ability and strong root activity in the later stage. The formulated fertilizer 14–7-6 (N-P_2_O_5_-K_2_O) was applied as the basal fertilization. The fertilizer was pelleted and did not contain other nutrients except N, P and K. The N source in the fertilizer was urea. Nanocarbon and nano calcium carbonate were used as nano fertilizer synergist, and the nanomaterials were produced by Beijing Deke DaoJin Science And Technology Co., Ltd. Specific technical parameters are shown in Table 1. During the nano fertilizer preparation, certain amounts of fertilizer and nano carbon or nano calcium carbonate were weighed and after that mixed in a reactor at a relatively uniform speed to obtain synergic fertilizer. Before sowing, the synergic fertilizer was evenly scatter on the soil surface and ploughed into the soil by tillage.

**Table 1:** Technical parameters of nano carbon and nano calcium carbonate

	Nano carbon	Nano calcium carbonate
Content(%)	99.9	99.9
Average particle size(nm)	40	20
Specific surface area(m^2^·g^−1^)	200	30–60
Particle shape	sphere	sphere
Appearance	black powder	white powder
Loose packing density(g·cm^−3^)	0.09	0.32

### Experimental design

The experiments were carried out in pot and field conditions. The field experiment was performed at the West-Coast Economic New Area experiment base of Qingdao Agricultural University (35.53°N,119.58°E). The soil type of the field experiment was sandy loam soil. The experiment was conducted using a randomized complete block design (RCBD), the size of each plot was 5 × 6 = 30 m^2^, and each treatment was repeated 3 times. The experiment set up 7 treatments, and the specific experimental design is shown in Table 3. T1, T2 and T3 treatments were set up to study the effects of different nano synergists and no nano synergist on N utilization and related gene expression in wheat. T4, T5 and T6 treatments were set up to study the effects of reducing fertilization after applying nano synergist. The first-year experiment was sown on October 11, 2018, with a seeding rate of 150 kg·ha^−1^, and harvested on June 12, 2019. The second-year experiment was sown on October 12, 2019, with a seeding rate of 127.5 kg·ha^−1^, and harvested on June 17, 2020. Planted corn in the field plot from the wheat harvest in the first year to the reseeding. The climatic data during the field experiment is shown in Table 2. During the whole growth period, the wheat crop did not receive irrigation and fertilizer topdressing. The experiment set up 7 treatments, and the specific experimental design is shown in Table 3. The pot experiment was carried out at the experimental base of Qingdao Agricultural University (36.30°N, 120.36°E). The experiment was conducted using a randomized block design, set up7 treatments, each treatment was repeated ten times, and ten wheat plants in each pot. The specific experimental design is shown in Table 4 and the soil nutrient content of field and pot is shown in Table 5. The Alkali hydrolyzed nitrogen in soil refers to 'mineral nitrogen (nitrate + ammonium)' and readily hydrolyzable organic nitrogen. The height of the pot was 30 cm, the diameter was 35 cm and the surface area was about 0.097 m^2^. The field soil was sieved, and after that, the pots were filled with it. Then the synergic fertilizer was applied on the soil and the seeds sowed there.

**Table2:** Weather conditions during the field experiment

	**2018–2019**		**2019–2020**	
Month	Average temperature (℃)	Average precipitation (mm)	Average temperature (℃)	Average precipitation (mm)
**Oct**	14.4	23.1	15.9	18.4
**Nov**	8.8	18.3	9.2	14.7
**Dec**	0.5	10.5	2.1	11.9
**Jan**	-1.2	7.6	-0.6	1.4
**Feb**	1.6	12.1	4	4
**Mar**	9.15	16.2	9.25	9.9
**Apr**	13.95	30	13.2	32.4
**May**	22.25	31.2	20.1	25.3
**Jun**	26	48.1	25.05	32.3

**Table 3:** Field experiment treatments

Treatment	Treatment Content	Content of Each Component		
		**Compound fertilizer** **(**kg·ha^−1^**)**	**Nano carbon synergist** (g·ha^−1^**)**	**Nano calcium carbonate synergist** (g·ha^−1^**)**
CK	compound fertilizer	525		
T1	compound fertilizer + 0.3%nano carbon synergist	525	1575	
T2	compound fertilizer + 0.3%nano calcium carbonate synergist	525		1575
T3	compound fertilizer + 0.3%composite nano synergist	525	787.5	787.5
T4	70%compound fertilizer + 0.3%nano carbon synergist	367.5	1102.5	
T5	70%compound fertilizer + 0.3%nano calcium carbonate synergist	367.5		1102.5
T6	70% compound fertilizer + 0.3% composite nano synergist	367.5	551.25	551.25

**Table 4:** Pot experiment treatments

Treatment	Treatment Content	Content of Each Component (g·dm^−3^)		
		**Compound fertilizer**	**Nano carbon synergist**	**Nano calcium carbonate synergist**
CK	compound fertilizer	0.175		
T1	compound fertilizer + 0.3% nano carbon synergist	0.175	5.26 × 10^–4^	
T2	compound fertilizer + 0.3% nano calcium carbonate synergist	0.175		5.26 × 10^–4^
T3	compound fertilizer + 0.3% composite nano synergist	0.175	2.63 × 10^–4^	2.63 × 10^–4^
T4	70% compound fertilizer + 0.3% nano carbon synergist	0.123	3.68 × 10^–4^	
T5	70% compound fertilizer + 0.3% nano calcium carbonate synergist	0.123		3.68 × 10^–4^
T6	70% compound fertilizer + 0.3% composite nano synergist	0.123	1.84 × 10^–4^	1.84 × 10^–4^

**Table 5:** Soil nutrient content

Experiment type	Years	Total nitrogen (mg·kg^−1^)	Alkali hydrolyzed nitrogen (mg·kg^−1^)	Available phosphorus (mg·kg^−1^)	Available potassium (mg·kg^−1^)	Organic matter (mg·kg^−1^)	pH
Field	2018	1.21 × 10^4^	92.2	35.4	161.3	1.22 × 10^4^	6.43
	2019	1.17 × 10^4^	90.5	33.9	157.1	1.24 × 10^4^	6.16
Pot	2018	1.14 × 10^4^	87.37	25.34	159.9	1.19 × 10^4^	6.37
	2019	1.16 × 10^4^	85.48	28.63	157.5	1.21 × 10^4^	6.23

Alkali hydrolyzed nitrogen refers to 'mineral nitrogen (nitrate + ammonium)' and readily hydrolyzable organic nitrogen

## Measurement items and methods

### Plant nitrogen content

Starting from the anthesis period and then sampling every 7 days, samples are taken on May 8, May 15, May 22, May 29, June 5, and June 12, 2019 in the first year, and on May 13, May 20, May 27, June 3, June 10, and June 17, 2020 in the second year. Five whole wheat plants were randomly selected from each plot in the field experiment, and five whole plants were randomly selected from each treatment in the pot experiment, and then separated into stems, leaves, spikes and roots, individually by plot. Samples of each part were thoroughly washed, and processed in an oven at 105 °C for 30 min, dried to constant weight at 80℃ and then weighed. After grinding, the sample was boiled according to the specified method, and the N content of each part was measured by Kjeldahl N analyzer [22, 23]. The N accumulation was calculated according to equation Eq. 1, and the relative N use rate was calculated according to Eq. 2 [24, 25].

1 $$\mathrm N\;\mathrm{accumulation}\;\left({\mathrm g\cdot\mathrm{plant}}^{-1}\right)=\mathrm{plant}\;\mathrm{dry}\;\mathrm{weight}\times\mathrm{plant}\;\mathrm N\;\mathrm{concentration}$$

2 $$\mathrm{Relative}\;\mathrm N\;\mathrm{use}\;\mathrm{rate}\;\left(\%\right)=\left(\mathrm N\;\mathrm{accumulation}-\mathrm{control}\;\mathrm N\;\mathrm{accumulation}\right)/\mathrm N\;\mathrm{application}\;\mathrm{amount}\times100$$

### Glutamine synthetase activity

Took a certain amount of fresh wheat leaves, cut them into pieces in the mortar, then added with phosphate buffer, ground and centrifuged, the supernatant was the crude enzyme extract. Added the enzyme reaction solution into it for reaction, and then added trichloroacetic acid (30%), 5.5 mol · L^−1^ hydrochloric acid and 8% ferric chloride mixed in a ratio of 1:1:1. After standing for a period of time, compared the color at 540 nm. Repeated 3 times and took the average [26].

### Gene expression

Took a certain amount of wheat fresh flag leaves, and used the SteadyPure plant RNA Extraction Kit (Accurate Biology, Changsha, China) for RNA extraction, and the specific operation was carried out according to the instructions. R223 reverse transcription kit (Vazyme Biotech Co.,Ltd, Nanjing, China) was used to obtain cDNA. Q341 RT-PCR reagent (Vazyme Biotech Co.,Ltd, Nanjing, China) was used for Fluorescence quantitative PCR with the following program: 95℃ for 30 s; 40 cycles of 95 ℃ for 5 s, 60 ℃ for 15 s, 72 ℃ for 20 s. The test genes were analyzed by the 2^−△△Ct^ method [27]. The gene and primer sequences were determined as shown in Table 6.

**Table 6:** Sequences of all primers used in quantitative real-time PCR

Gene	Primer sequence	Product size(bp)
*TaGS1*	F:CAAGTGGAACTACGACGGCT	481
	R:GCCAGCAGAAATGCCAAC	
*TaGS2*	F:TGACAGGGCTACACGAGACA	311
	R:ACGAACAGCCTCGCCAC	
*TaNRT2.2*	F:CGGTTCCTCATTGGCTTCT	295
	R:TCGCAAGGTTCCCGTCAG	
*TaNRT2.3*	F:TGCCATCCAAAAGTGCGGT	395
	R:CGCCAAGGTCAGAGAGGT	

### Data processing

The experimental data were collected using Excel 2016. The variance analysis and difference significance test (*P* < 0.05) were analyzed using SPSS 24 software [28], Duncan's new multiple range method was used for multiple comparisons.

## Results and analysis

### Effects of nano fertilizer synergists on nitrogen utilization in wheat

#### Effect of nano fertilizer synergist on nitrogen accumulation in wheat

**Fig. 1:**
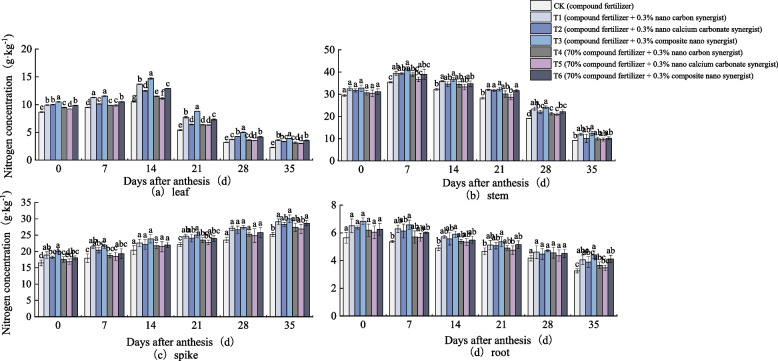
Nitrogen concentration in (**a**) leaf, (**b**) stem, (**c**) spike, and (**d**) root of wheat under different treatments (2018–2019 field). The type of mean comparison test was Duncan's new multiple range method. The vertical bar represents the standard error, and the different letters above the error line represent the significant difference in the mean values of different treatments of the same measurement item (*P* < 0.05)

Figure 1 shows the variation trend of N concentration in each part of wheat plants in the field is different. The N concentration in wheat leaves increased first and then decreased with the increase of days after anthesis. The N concentration in leaves was the highest at 14 days after anthesis (Fig. 1a). At this time, there was a significant difference in the N concentration in leaves of all treatments. The N concentration of T3 was the highest, and the N concentration of all treatments was significantly higher than CK, while the difference in other periods was relatively small. With the increase of days after anthesis, the N concentration in wheat stem also increased first and then decreased, and reached the highest value 7 days after anthesis (Fig. 1b). At this time, the N concentration in T3 stem was the highest, followed by T1, T2 and T6, and the N concentration of CK was the lowest. The N concentration in wheat spike increased gradually with the increase of anthesis days, and the N concentration of CK was lower than that of other treatments (Fig. 1c). The N concentration in each treatment spike was T3 > T1 > T6 > T2 > T4 > T5 > CK. The N concentration in wheat roots decreased gradually in the growth process after wheat anthesis (Fig. 1d). The N concentration of T3 and T6 was still higher than that of other treatments to a certain extent, and the N concentration of CK was lower than that of other treatments.

**Fig. 2:**
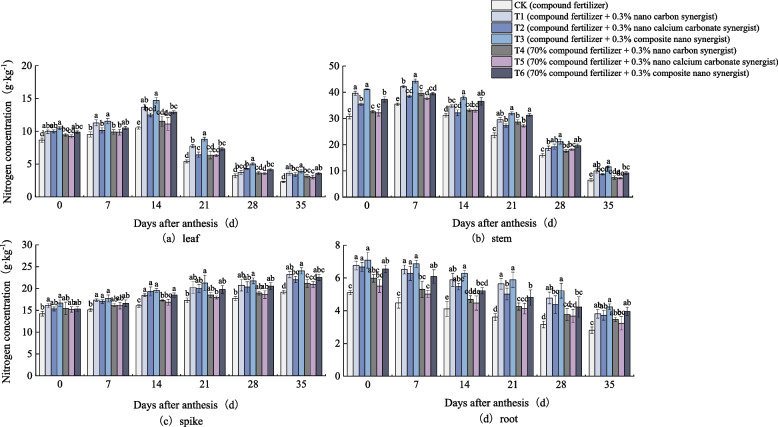
Nitrogen concentration in (**a**) leaf, (**b**) stem, (**c**) spike, and (**d**) root of wheat under different treatments (2019–2020 pot). The type of mean comparison test was Duncan's new multiple range method. The vertical bar represents the standard error, and the different letters above the error line represent the significant difference in the mean values of different treatments of the same measurement item (*P* < 0.05)

It can be seen in Fig. 2 that the N concentration in each part of potted wheat plants shows different trends. In the pot experiment, the N concentration in wheat leaves increased first and then decreased with the increase of days after anthesis (Fig. 2a). At 14 days after anthesis, the N concentration in leaves of all treatments reached the highest, and then began to decrease gradually. At this time, there was a significant difference in the N concentration of leaves of all treatments, T3 was significantly higher than other treatments, CK was significantly lower than other treatments. In the growth process of 14–35 days after anthesis, the decrease range of N concentration in leaves of all treatments was CK > T1 > T3 > T2 > T5 > T4 > T6, and the decrease range of CK was the largest. The N concentration in wheat stem first increased and then decreased with the increase of anthesis days, and reached the highest at 7 days after anthesis (Fig. 2b). At this time, the N concentration of T3 was the highest, which was significantly higher than that of other treatments. In the period of 0–35 days after anthesis, the N concentration in stem of CK was significantly lower than that of other treatments. The N concentration in wheat spike increased gradually with the increase of days after anthesis, and the N concentration of T3 and T6 remained at a high level (Fig. 2c). In the period of 0–35 days after anthesis, the N concentration of CK was still lower than that of other treatments. The N concentration of wheat roots decreased gradually with the increase of days after anthesis (Fig. 2d). The decline range of N concentration of roots in each treatment was as follows: CK > T2 > T1 > T4 > T5 > T3 > T6. The N concentration of CK was significantly lower than that of other treatments, and the decline range was the largest.

It can be seen that in the field experiment and pot experiment, the variation of N concentration in each part of wheat are basically the same (Figs. 1 and 2).

**Fig. 3:**
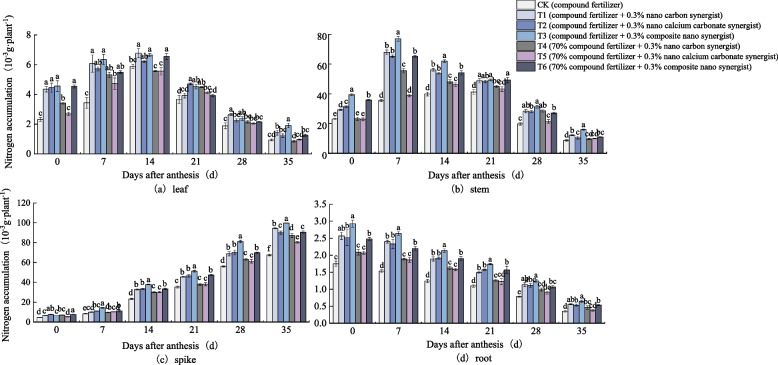
Nitrogen accumulation in (**a**) leaf, (**b**) stem, (**c**) spike, and (**d**) root of wheat under different treatments (2018–2019 field). The type of mean comparison test was Duncan's new multiple range method. The vertical bar represents the standard error, and the different letters above the error line represent the significant difference in the mean values of different treatments of the same measurement item (*P* < 0.05)

**Fig. 4:**
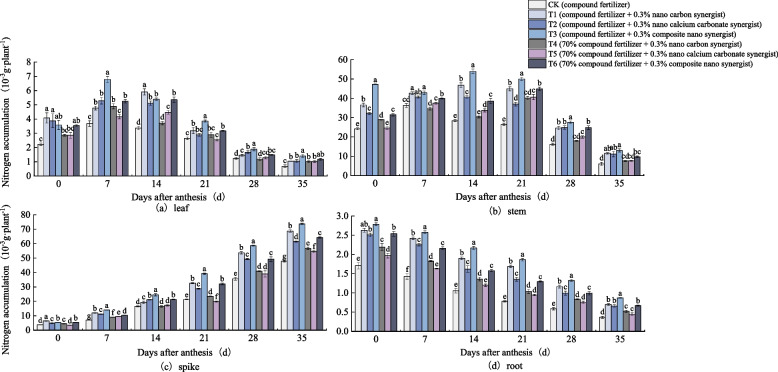
Nitrogen accumulation in (**a**) leaf, (**b**) stem, (**c**) spike, and (**d**) root of wheat under different treatments (2019–2020 pot). The type of mean comparison test was Duncan's new multiple range method. The vertical bar represents the standard error, and the different letters above the error line represent the significant difference in the mean values of different treatments of the same measurement item (*P* < 0.05)

As shown in Fig. 3, in the field experiment, the N accumulation in wheat leaves and stems showed a trend of first increasing and then decreasing. The N accumulation in leaves reached the maximum on 14 days after anthesis, and the N accumulation in stems reached the maximum on 7 days after anthesis (Fig. 3a and b). And the N accumulation in T1-T6 treatments with nano synergist was higher than CK in wheat stems and leaves. The N accumulation in wheat spikes showed a trend of gradual increase (Fig. 3c). From 7 days after anthesis, the N accumulation in spikes of T1-T6 treatments was significantly higher than CK and on 35 days after anthesis, the N accumulation of T1-T6 treatments was 39.7%, 33.3%, 47.8%, 29.1%, 19.1%, 34.1% higher than CK respectively. The N accumulation in wheat roots decreased gradually (Fig. 3d). It can be seen in Fig. 3a, b, c and d that the N accumulation of each part of wheat in the late anthesis period was significantly higher in the T3 and T6 treatments applying the composite nano synergist than in the T1, T2, T4 and T5 treatments applying the nano carbon or nano calcium carbonate synergist, and even under the condition of reduced fertilization, the N accumulation of the T4, T5 and T6 treatments applying the nano synergist is still significantly higher than CK. As shown in Fig. 4, the trend of N accumulation of each treatment in the pot experiment was basically the same as that in the field experiment.

#### Effect of nano fertilizer synergist on relative N use rate of wheat

As shown in Table 7, in the field and pot experiments, the relative N use rate of the treatments with nano synergist was significantly higher than that of CK under both normal fertilization and fertilizer reduction conditions, and the relative N use rate of T3 and T6 treatments with compound nano synergist was respectively higher than that of T1, T2 and T4, T5 treatments with only nano carbon synergist or nano calcium carbonate synergist. The relative N use rate of the treatments with nanocarbon synergist was higher than that of the treatments with nano calcium carbonate synergist. The results showed that the application of nano synergist could significantly improve the relative N use rate of plants, and the effect of compound nano synergist was the best.

**Table 7:** Relative N use rate of wheat of different treatments

Treatment	Nitrogen accumulation		Relative N use rate (%)	
	Field(kg·ha^−1^)	Pot (g·pot^−1^)	Field	Pot
CK (compound fertilizer)	282 d	0.56c	0	0
T1 (compound fertilizer + 0.3% nano carbon synergist)	322.65 ab	0.83ab	18.87	12.90
T2 (compound fertilizer + 0.3% nano calcium carbonate synergist)	318.9 bc	0.75ab	17.14	9.08
T3 (compound fertilizer + 0.3% composite nano synergist)	327.45 a	0.90a	21.13	16.25
T4 (70% compound fertilizer + 0.3% nano carbon synergist)	318.15 bc	0.67bc	16.81	5.26
T5 (70% compound fertilizer + 0.3% nano calcium carbonate synergist)	314.7 c	0.65bc	15.20	4.30
T6 (70% compound fertilizer + 0.3% composite nano synergist)	321.63 ab	0.77ab	18.4	10.04

The type of mean comparison test was Duncan's new multiple range method. The different letters represent the significant difference in the mean values of different treatments of the same measurement item (*P* < 0.05)

### Effects of nano fertilizer synergist on the expression of genes related to nitrogen transport and metabolism in wheat

#### Effects of nano fertilizer synergists on gene expression of nitrate transporter

**Fig. 5:**
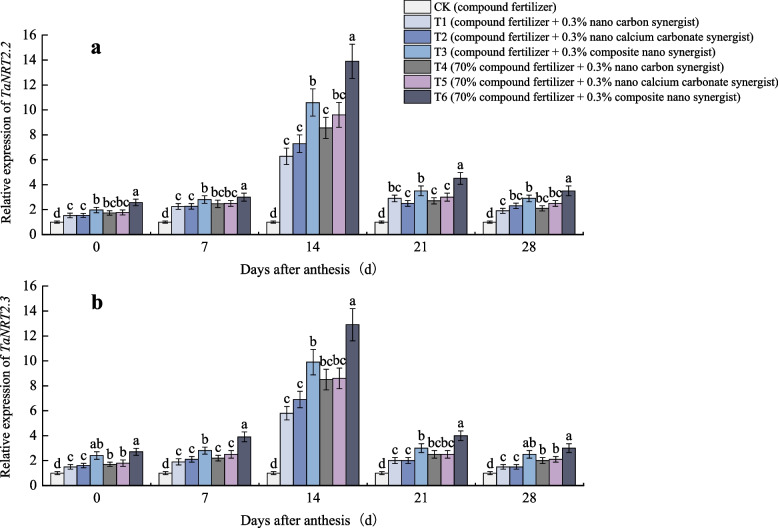
Relative expression of (**a**) *TaNRT2.2* and (**b**) *TaNRT2.3* of different treatments. The type of mean comparison test was Duncan's new multiple range method. The vertical bar represents the standard error, and the different letters above the error line represent the significant difference in the mean values of different treatments of the same measurement item (*P* < 0.05)

The above results showed that nano fertilizer synergists have a significant effect on the N content of wheat plants. Therefore, it is speculated whether nano fertilizer synergists affect the N accumulation of wheat plants by affecting the N transport and metabolism process in wheat plants. In this experiment, the variation in the expression of wheat plant related genes in the field experiment was measured. As shown in Fig. 5, after the application of nano fertilizer synergist, the relative expression of *TaNRT2.2* was up-regulated during the grain filling period. The relative gene expression levels of *TaNRT2.2* and *TaNRT2.3* were the highest at 14 days after anthesis. The two relative gene expression levels of T6 were significantly higher than those of other treatments at all stages after anthesis. Under the condition of reducing the amount of fertilization application, the relative expression level of nitrate transporter gene in each treatment was significantly higher, and the effect of applying composite nano fertilizer synergist was the best. The trend of N content is consistent with the above results.

#### Effects of nano fertilizer synergist on glutamine synthase activity and gene expression

**Fig. 6:**
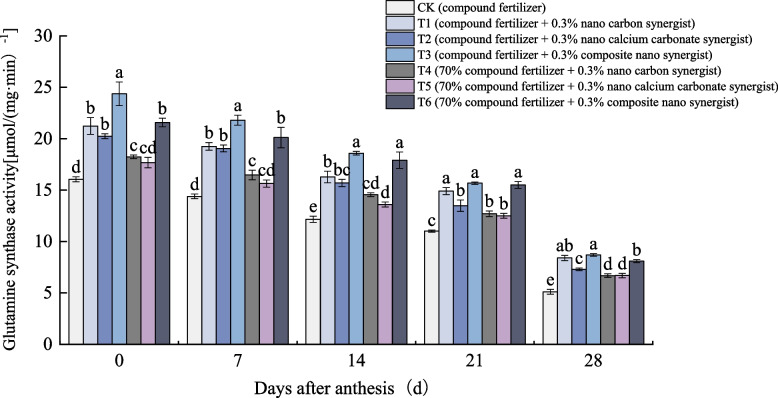
Glutamine synthase activity of different treatments. The type of mean comparison test was Duncan's new multiple range method. The vertical bar represents the standard error, and the different letters above the error line represent the significant difference in the mean values of different treatments of the same measurement item (*P* < 0.05)

It can be seen from Fig. 6 that the glutamine synthase (GS) activity of flag leaves of wheat in each treatment decreased gradually 0–28 days after anthesis. The treatment with nano fertilizer synergist was significantly higher than CK, and the GS activity of the treatment with reduced fertilization was always higher, and the GS activity of the treatment with composite nano fertilizer synergist was significantly higher than that of other treatments. The decline rate of GS activity in wheat flag leaves was slower 0–21 days after anthesis, and the decline rate of GS activity in wheat flag leaves was faster 21–28 days after anthesis.

**Fig. 7:**
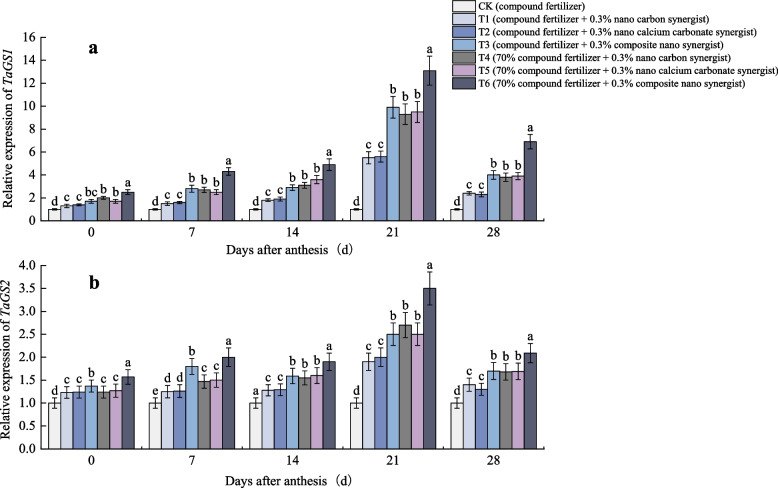
Relative expression of (**a**) *TaGS1* and (**b**) *TaGS2* of different treatments. The type of mean comparison test was Duncan's new multiple range method. The vertical bar represents the standard error, and the different letters above the error line represent the significant difference in the mean values of different treatments of the same measurement item (*P* < 0.05)

According to the variation of GS activity in flag leaves, it is speculated whether nano fertilizer synergist can regulate wheat N metabolism by affecting wheat GS synthesis gene. As shown in Fig. 7, the relative expression level of *TaGS1* and *TaGS2* reached the highest 21 days after anthesis. At each stage after anthesis, the relative expression level of genes in each treatment was the highest in T6, and there was no significant difference between T3, T4 and T5. The relative expression level of genes under reduced fertilization was higher than that under conventional fertilization.

## Discussion and conclusion

### Analysis of the effect of nano fertilizer synergist on nitrogen utilization of wheat

Plant growth is affected by many factors. In addition to external factors such as light and temperature, N as an essential nutrient also plays an important role in plant growth and development. Nitrogen is not only an important component of plant hormone and chlorophyll, but also the main component of protein and nucleic acid. It is essential in the process of plant growth [29]. Wang Guodong et al. [30] found that the application of nano carbon could promote the absorption and utilization of N by strawberry (*Fragaria* × *ananassa Duch.*) plants, and the soil N residue is significantly increased and the N loss is significantly reduced. This may be due to the surface effect and small-scale effect of nano carbon, which increases the adsorption performance of fertilizer, as well as reduces the leaching loss of fertilizer. The study also found that the application of nano carbon significantly increased the absorption and transportation of N in each organ of strawberry and promoted the accumulation and partition of N in each organ. Liang Taibo et al. [31] found that the N accumulation of each organ of fluecured tobacco (*Nicotiana tabacum L.*) treated with nano carbon fertilizer increased at maturity, and the N utilization rate was significantly higher than that of the control treatment. Li Shumin et al. [32] found that adding nano carbon synergist can significantly promote the growth of maize (Zea mays L.), improve the absorption and utilization of N, and save fertilizer significantly. In this experiment, after applying nano fertilizer synergist, the N concentration in all parts of wheat plant increased significantly. According to the N concentration in wheat leaves that reached the highest at 14 days, and the N concentration of stems that reached the highest at 7 days after anthesis, it can be assumed that N transport from roots to aboveground stems and leaves was gradually, and finally accumulated in wheat spike. Nano carbon is a kind of non-conductive modified carbon, which can absorb N from ammonium ions, release hydrogen ions at the same time, and reduce the loss of ammonium N in soil [33]. Nitrate accounts for a large proportion of soil N, which is the main form of N loss [34]. Adding nano carbon to soil inhibits the activity of nitrifying bacteria and reduce the content of NO_3_^−^ [35], and then reduce the loss of N. In this experiment, the application of nano carbon, nano calcium carbonate and composite nano fertilizer synergist effectively improved the N accumulation. At 35 days after wheat anthesis, under normal fertilization conditions, the N accumulation of T3 was significantly higher than that of T1 and T2; Under the condition of reducing the amount of fertilization, the N accumulation of T6 was higher than that of T4 and T5; The N accumulation of the treatment with nano fertilizer synergist was significantly higher than that of CK. It shows that the application of composite nano fertilizer synergist is more conducive to the transport and accumulation of N in wheat plants. After the application of nano carbon and nano calcium carbonate, the N utilization rate was significantly higher than that of CK. Under the condition of reducing the amount of fertilization, the effect of composite nano fertilizer synergist was the best, indicating that nano carbon and nano calcium carbonate after mixing with fertilizer can significantly promote the absorption and utilization of soil N by wheat plants.

### Analysis of the effect of nano fertilizer synergist on the expression of genes related to nitrogen absorption in wheat

The N level in wheat plants is affected by many processes such as N transport and metabolism. According to the difference of N content in each part of wheat plant, this experiment measured whether there was difference in the relative expression of relevant regulatory genes. Nitrate transporter gene *TaNRT2.2* and *TaNRT2.3* plays an important role in N transport [36]. Nitrate and NH_4_^+^ are the inorganic N sources for plant absorption and utilization, and NO_3_^−^ is the main N form for plant absorption and utilization [37], which requires NO_3_^−^ transporter for transport. Xuan hongmei et al. [38] found that NO_3_^−^ transporters play an important role in the response of wheat seedlings to N starvation under the condition of N starvation, and speculated that NO_3_^−^ transporter genes can improve the utilization efficiency of plants for NO_3_^−^. By detecting the relative expression of NO_3_^−^ transporter gene, it was found that the relative expression of each treatment was the highest 14 days after wheat anthesis, and the accumulation of N in leaves reached the highest at the same time, indicating that the application of nano fertilizer synergist promoted the up-regulation of NO_3_^−^ transporter gene, which was beneficial to the accumulation of N in wheat leaves. Through N assimilation, GS can use the energy released by ATP decomposition to catalyze the inorganic N in chemical fertilizer into organic N such as glutamine and glutamic acid for plant utilization, which is the key enzyme of N metabolism in plants [39]. Thus, GS plays a central role in N metabolism, participates in the regulation of various N metabolism [40], and plays an important role in the process of plant N assimilation and metabolism [41]. The high expression of GS can improve the N use efficiency of wheat plants [42]. Meng Weiwei et al. [43] found that GS activity of wheat is closely related to N content. With the appropriate increase of N content, GS activity of wheat flag leaves increases, which is conducive to improving NH_4_^+^ assimilation efficiency. Li Jing et al. [44] also found that the GS activity of wheat increased with the increase of N content, which was conducive to improving the protein content of wheat grains. In this experiment, after applying nano fertilizer synergist, GS activity was significantly improved under normal fertilization conditions and reduced fertilization. According to the difference of GS activity, there are also relevant differences in the relative gene expression of *TaGS1* and *TaGS2* [45, 46]. The relative gene expression of T6 was the highest in each period, indicating that the application of composite nano fertilizer synergist under the condition of reducing the amount of fertilization was more conducive to promote the metabolism of N in wheat plants. In addition, AMT gene family plays a certain role in NH_4_^+^ transport, and also directly participates in nitrogen absorption pathway [47]. And during N assimilation, NO_3_^−^ is reduced to NO_2_^−^ by NR and then reduced to NH_4_^+^ by NiR, and then NH_4_^+^ is assimilated to Gln / Glu by GS / GOGAT [48]. The expression of NR, NiR and GOGAT related genes also plays an important role in the absorption and utilization of N in wheat, which will be further verified in future research.

In conclusion, the results of this experiment showed that the application of nano fertilizer synergist can induce the expression of genes related to N transport and metabolism, as well as to promote N accumulation in wheat plants and improve wheat yield.

## Data Availability

The datasets used and/or analysed during the current study are available from the corresponding author on reasonable request.
